# Prognostic role of *Helicobacter pylori* infection in acute coronary syndrome: a prospective cohort study

**DOI:** 10.5830/CVJA-2011-016

**Published:** 2012-04

**Authors:** Rahimeh Eskandarian, M Madani, R Ghorbani, M Shiyasi, B Momeni, K Hajifathalian

**Affiliations:** Department of Cardiology, Faculty of Medicine, Seman, University of Medical Sciences, Semnan Iran; Department of Cardiology, Faculty of Medicine, Seman, University of Medical Sciences, Semnan Iran; Department of Community Medicine, Faculty of Medicine, Semnan University of Medical Sciences, Semnan, Iran; Department of Community Medicine, Faculty of Medicine, Semnan University of Medical Sciences, Semnan, Iran; Department of Community Medicine, Faculty of Medicine, Semnan University of Medical Sciences, Semnan, Iran; Department of Community Medicine, Faculty of Medicine, Semnan University of Medical Sciences, Semnan, Iran

**Keywords:** H *pylori*, acute coronary syndrome, prognosis

## Abstract

**Abstract:**

In a prospective cohort study, we evaluated the effect of *Helicobacter pylori* seropositivity on the risk of future adverse cardiovascular outcomes among patients with acute coronary syndrome (ACS). In 433 patients, IgA and IgG antibodies to H *pylori*, along with classic risk factors, including hypertension, diabetes, hyperlipidaemia, smoking and family history of coronary artery disease (CAD) were determined. Short-and long-term follow-up information on adverse outcomes, defined as recurrence of unstable angina, myocardial infarction, coronary angioplasty, coronary artery bypass graft surgery, and sudden cardiac death was obtained. None of the classic CAD risk factors correlated with incidence of either short- or long-term outcomes. Seropositivity for H *pylori* was significantly associated with risk of short-term adverse outcomes, and independently predicted their incidence in multivariate regression (*R* = 3.05, *p* < 0.001). Results failed to show such an association between H *pylori* seropositivity and long-term adverse outcomes. H *pylori* infection may affect short-term prognosis in patients with ACS. Randomised trials are needed to evaluate the role of H *pylori* eradication in these patients.

Coronary artery disease (CAD) is the leading cause of death in developed and transitioning countries.^1^ Classic CAD risk factors, such as dyslipidaemia, hypertension, smoking, family history of CAD, and diabetes mellitus have not been able to fully explain the variations in CAD incidence, morbidity and mortality,^2^,^3^ and there is a need to search for possible new causal mechanisms affecting pathogenesis and prognosis of CAD.^4^

Certain bacterial and viral pathogens have been suggested to play a role in development and/or prognosis of CAD, including *Chlamydia pneumonia, Helicobacter pylori*, cytomegalovirus, Coxsackie virus, Hepatitis A virus and *Herpes simplex* virus.^5^-^7^ Numerous studies, mostly cross-sectional or case-control studies, have assessed the association between *H pylori* infection and CAD.^8^-^11^ Various mechanisms have been proposed to explain the role of *H pylori* infection in the pathogenesis of CAD, namely causing persistent local or systemic inflammation, and initiating autoimmune responses.^12^,^13^ However, studies provide discordant data on the association between *H pylori* infection and CAD.

While some studies report a significant relationship between the two,^14^-^18^ others suggest no, or at best a weak association between *H pylori* infection and CAD.^19^-^23^ As a result of this discrepancy, the actual role of *H pylori* in the pathogenesis and prognosis of CAD has remained largely controversial,^24^-^26^ and many authors suggest well-designed prospective studies to further investigate the association between *H pylori* and CAD.^27^,^28^ Moreover, *H pylori* diagnosis is fairly simple and its treatment much easier and less costly, compared to that of classic CAD risk factors.^4^ Therefore finding a causal relationship between *H pylori* infection and prognosis in patients with CAD, especially patients presenting with acute coronary syndrome (ACS), may enable clinicians to decrease morbidity and mortality simply by treating *H pylori* infection in these patients.^18^

In the light of this, the present study was designed as a prospective cohort to investigate the effect of current *H pylori* infection on short- and long-term prognosis in patients presenting with ACS. The results of this study will help to clarify the relationship between *H pylori* infection and CAD. In addition, it will provide information regarding the possible role of *H pylori* eradication in patients presenting with ACS.

## Methods

This was a prospective cohort study carried out at the Fatemieh Hospital, Semnan, Iran. Between January 2004 and November 2006, a total of 450 patients admitted for ACS to the emergency ward or critical care unit agreed to participate in the study and were enrolled. Patient selection was done by the census sampling method. The objective and necessary procedures were clearly explained to these patients and all participants provided informed written consent before enrollment. The study was approved by the medical ethics committee of the hospital.

ACS (inclusion criteria) was defined as presenting with either unstable angina (clinical diagnosis) or myocardial infarction (MI), defined as significant ST elevation or new left bundle branch block in electrocardiography or increased levels of cardiac enzymes. During the one-year follow-up period, 17 participants were excluded from the study due to either receiving treatment for *H pylori* infection or becoming unavailable for follow up.

Risk factors evaluated in this study were: hypertension, diabetes mellitus (DM), hyperlipidaemia, cigarette smoking, and family history of CAD. Hypertension was defined as arterial blood pressure ≥ 140/90 mmHg or being treated with antihypertensive drugs. DM was defined as fasting blood sugar levels ≥ 126 mg/dl or being treated for this diagnosis with either diet or medication. Hyperlipidaemia was defined as low-density lipoprotein (LDL) cholesterol levels ≥ 130 mg/dl or being treated with lipid-lowering medication. Patients were considered to be smokers if they were current smokers or had stopped smoking less than three years before enrollment. Positive family history of CAD was considered in patients with a history of at least one first-degree relative affected by CAD before the age of 55 and 65 years for male and female relatives, respectively.

The main outcome evaluated in this study was recurrence of a cardiovascular event, defined as recurrence of unstable angina (UA), MI, performing coronary angioplasty during the study period, coronary artery bypass graft (CABG) surgery, or sudden cardiac death (SCD). Data regarding these outcomes were collected when patients were visited at pre-scheduled one-month and one-year intervals from their enrollment, to be evaluated for short- and long-term prognosis, respectively. When patients did not attend their appointments, they or their families were contacted by telephone. If the participant had died, the cause of death was determined by taking a history from family members and reviewing medical documents. Otherwise participants were asked to attend an appointment for follow up.

The main independent variable evaluated in the study was *H pylori* infection, defined as seropositivity for anti-*H pylori* antibodies. Both IgG and IgA antibodies against *H pylori* were tested for each participant at enrollment, and a patient was considered seropositive if positive for either IgA or IgG antibodies.

All participants were enrolled on their first day of admission for ACS, and all blood samples were collected the next morning and tested the same day. *H pylori* IgG and IgA antibodies were determined using commercially available ELISA assays (RADIM kit, Milan, Italy). According to the kit’s reference values, levels of more than 30 and less than 15 units? were considered positive and negative, respectively. Levels between these values were considered borderline. A total of 10 participants had borderline levels for *H pylori* IgA, and were designated as having a negative result. At enrollment and during each follow-up visit, patients underwent a complete evaluation, including a physical examination, electrocardiography, and review of all paraclinical data and medical history pertaining to study outcomes and/or *H pylori* eradication.

## Statistical analysis

Data are presented as number (percent) or mean (± SD). Possible association between categorical variables was assessed using the χ2 test. Relative risk was calculated to estimate the increase in risk of incidence of outcomes among *H pylori* seropositive patients, compared to seronegative participants. Logistic regression models were used to identify significant determinants of incidence of outcomes. All statistical tests used are reported with two-tailed estimates of type I error (*p*-value); *p* < 0.05 was considered significant. All statistical analysis was done using SPSS, version 13.0 (SPSS Inc, Chicago, USA).

## Results

A total of 450 subjects were enrolled in the study, of whom 433 completed the study and were followed up for one year. Seventeen patients were excluded either because of *H pylori* eradication during the study period or becoming unavailable for follow up. Patients’ ages ranged between 29 and 85 years, with a mean of 60.9 (± 12.3) years. Of 433 patients, 245 (56.6%) were male. Of all the participants, 204 patients (47.1%) were seropositive for *H pylori*. Of the whole study population, 69 (15.9%) patients developed short-term outcomes, defined as being diagnosed with UA or MI, or undergoing angioplasty, CABG or SCD during the first month after enrollment; 194 (44.8%) participants developed long-term outcomes, defined as occurrence of the same conditions mentioned above during the one-year follow-up period.

Table 1 summarises the prevalence of the five evaluated classic risk factors among the participants at the time of enrollment. Hypertension had the highest prevalence, as 221 (51.0%) patients were hypertensive, and DM showed the lowest prevalence, as only 109 (25.1%) subjects were diabetic.

**Table 1:** Prevalence Of Classic Cad Risk Factors Among Participants, Incidence Of Adverse Outcomes In Patients With And Without Each Risk Factor, And Distribution Of *H Pylori* Seropositivity Across Risk Factors

		*Total study population (n = 433) n (%)*	*Patients with short-term outcomes n (%)*	*Patients with long- term outcomes n (%)*	*Patients with positive antibody to H pylori n (%)*
Hypertension	+	221 (51.0)	33 (14.9)	100 (45.2)	106 (47.9)
	-	212 (49.0)	36 (16.9)	94 (44.3)	98 (46.2)
Diabetes	+	109 (25.2)	19 (17.4)	55 (50.5)	51 (46.7)
	-	324 (74.8)	50 (15.4)	139 (42.9)	153 (47.2)
Smoking	+	137 (31.6)	20 (14.6)	55 (40.1)	63 (45.9)
	-	296 (68.4)	49 (16.5)	139 (46.9)	141 (47.6)
Hyperlipidaemia	+	126 (29.1)	22 (17.4)	53 (42.1)	57 (45.2)
	-	307 (70.9)	47 (15.3)	141 (45.9)	147 (47.9)
Family history of CAD	+	111 (25.6)	15 (13.5)	53 (47.7)	49 (44.1)
	-	322 (74.4)	54 (16.7)	141 (43.8)	155 (48.1)

Table 1 presents the number and percentage of patients with each risk factor who ultimately developed short- and long-term outcomes. When evaluated by χ2 test, the rate of short- and longterm outcomes among patients with and without each risk factor did not differ significantly (*p* > 0.05). In other words, none of the classic risk factors including hypertension, DM, smoking, hyperlipidaemia and family history of CAD were associated with occurrence of cardiovascular events during the study period. In addition, the classic risk factors did not show any association with *H pylori* seropositivity, as similar numbers of patients with or without each risk factor were positive for *H pylori* antibodies (*p* > 0.05).

To evaluate *H pylori* infection as a possible risk factor, the rate of developing outcomes was compared between seropositive and seronegative patients. As shown in Table 2, of 204 seropositive patients, in 48 (23.5%) subjects a cardiovascular event (as defined earlier) occurred during the first month after enrollment, compared to only 21 (9.1%) patients who developed short-term outcomes among 229 seronegative participants (*p* < 0.000). *H pylori*-infected patients were more than 2.5 times more likely to develop short-term adverse outcomes, as defined in this study (risk ratio: 2.58).

**Table 2:** Incidence Of Short- And Longterm Adverse Outcomes Based On Patients’ Seropositivity For *H Pylori* Iga And/Or Igg. Data Presented As Number (Percent)

		*Total study population (n = 433) n (%)*	Patients with short-term outcomes* n (%)	*Patients with long-term outcomes n (%)*
*H pylori*	+	204 (47.1)	48 (23.5)	97 (47.5)
IgA and/or IgG	–	229 (52.9)	21 (9.1)	97 (42.4)

*For χ^2^ test, *p* < 0.000.

*H pylori* seropositivity did not show any significant association with long-term adverse outcomes during the one-year follow up of patients; 97 (47.5%) patients among the 204 *H pylori*-positive participants developed long-term adverse outcomes, compared to 97 (42.5%) of 229 subjects who were negative for *H pylori* antibodies (*p* > 0.05). Seropositive patients were only marginally more likely to develop long-term adverse outcomes compared to their seronegative counterparts (risk ratio: 1.12).

When short-term adverse outcomes were compared individually between seropositive and seronegative participants, the results showed a significantly higher incidence of UA, MI, coronary angioplasty and SCD during the first month of follow up among *H pylori*-infected patients (*p* < 0.01). However, eight (3.4%) patients among the seronegative participants underwent CABG surgery during the first month, compared to four (1.9%) in the seropositive group (*p* < 0.01). Regarding longterm adverse cardiovascular outcomes, none of the individual events, including UA, MI, angioplasty, CABG and SCD differed between seronegative and seropositive participants (*p* > 0.05).

To evaluate the effect of risk factors on incidence of adverse cardiovascular events, a multivariate logistic regression model was built using age, gender and classic risk factors, including hypertension, DM, hyperlipidaemia, smoking and positive family history for CAD, in addition to seropositivity for *H pylori*. From all these risk factors, only *H pylori* infection proved to be a significant and independent predictor of short-term adverse cardiovascular outcomes (*R* = 3.05, *p* < 0.001). When this model was used for long-term outcomes, only age was a significant determinant of incidence of cardiovascular events (*R* = 1.04, *p* < 0.001).

## Discussion

In this study, we prospectively evaluated the effects of *H pylori* seropositivity and classic cardiovascular risk factors on incidence of future cardiovascular events among a cohort of 433 patients presenting with ACS. The main new finding of this study was to show a positive association between *H pylori* seropositivity and incidence of short-term adverse cardiovascular events in participants during the first month after presenting with ACS. However, we failed to provide evidence for such an association with long-term adverse outcomes during one year of follow up. Furthermore, in this study we did not find any significant relationship between *H pylori* seropositivity and any of the studied risk factors.

Some viral and bacterial infections, such as *Chlamydia pneumonia*, Hepatitis A virus, cytomegalovirus and *Herpes simplex* virus have been implicated in affecting development and course of coronary atherosclerotic diseases.^29^ *H pylori* is similar to these pathogens as it is also an obligatory intracellular pathogen and causes life-long persistent infection.^30^ *H pylori* also establishes persistent antibodies targeted to it.

Several pathological mechanisms have been postulated to explain the effect of *H pylori* infection on atherosclerosis. It has been suggested to initiate an acute-phase response and to activate TNF-α, IL-6 and fibrinogen, which are inflammatory cytokines that can directly or indirectly propagate an inflammatory process in arterial walls.^8^,^31^ *H pylori* has been also shown to cause platelet aggregation, an important aspect of acute destabilisation of atherosclerotic disease.^32^ Another possible mechanism by which *H pylori* may cause endothelial damage is by causing aggravated autoimmune hormonal responses because of antigenic mimicry,^13^ such as immunological cross reactivity between bacterial and human heat-shock proteins,^33^ which can lead to coronary calcification and early atherosclerosis.^34^ In addition, direct colonisation of the arterial wall by *H pylori* has been suggested,^21^ and *H pylori* has been found in atheromas using the polymerase chain reaction (PCR) technique.^35^

We measured both *H pylori* IgG and IgA antibodies in the participants. IgA and IgG seropositivity were measured in this study, as it has been suggested that IgA seropositivity may reflect more recent and active infection,^36^ and its use may be appropriate to evaluate the association between *H pylori* infection and ischaemic heart disease.^37^

In our study, we failed to show any significant association between presence of the classic cardiac risk factors, including hypertension, DM, hyperlipidaemia, smoking and positive family history of CAD, and incidence of future adverse cardiovascular outcomes. This was consistent with the results reported by Zhu *et al.*, who also did not demonstrate a prognostic role for these classic risk factors on future adverse cardiovascular events in their study on 890 patients with CAD.^38^ This may have been partly due to extensive treatment of these risk factors among patients diagnosed with CAD, which could have lessened their observed prognostic effects in the study population. Therefore, longer follow-up duration on larger sample populations may be needed to document the prognostic effects of these factors.

It has previously been suggested that there may be a relationship between the classic CAD risk factors and *H pylori* infection, which could explain the association between *H pylori* infection and CAD.^39^,^40^ However, this study did not demonstrate any significant association between *H pylori* seropositivity and the presence of classic risk factors. Our results are in agreement with those of Danesh *et al.*, who did not find such an association to be present in their meta-analysis of 18 different studies.^41^

Interestingly, results from the present study exhibited a positive association between *H pylori* infection and short-term adverse cardiovascular outcomes. Patients who were seropositive for *H pylori* at enrollment showed a higher risk for incidence of adverse cardiovascular events during the first month of follow up. They were 2.58 times more likely to be diagnosed with recurrence of UA, MI or SCD, or to undergo coronary angioplasty or CABG one month after presenting with ACS, compared to their seronegative counterparts. Additionally, in the logistic regression model used to predict incidence of short-term outcomes, which also included age, gender and classic risk factors, only *H pylori* infection proved to be a significant determinant of these events.

Previous studies report conflicting data regarding the relationship between *H pylori* infection and incidence of future cardiovascular events. Some studies did not find any association,^42^,^43^ whereas, others revealed a significant association.^44^,^45^ This discrepancy may be due to differences in the study populations. For example, some studies enrolled healthy adult subjects, while others assessed cohorts of patients with CAD. Furthermore, most of the previous studies measured only *H pylori* IgG antibodies,^43^,^46^ while in our study, we used both IgG and IgA antibodies to detect *H pylori* infection. In a previous study by Rupprecht *et al.*,^44^ while there was no relationship between *H pylori* IgG seropositivity and risk of fatal cardiovascular events, IgA seropositivity was significantly associated with fatal cardiovascular events, with a hazard ratio of 2.5, which is similar to our results.

Most importantly, in this study *H pylori* infection was only linked to short-term adverse outcomes, such as UA, MI and SCD, and did not show a significant long-term association with the same outcomes during the one year of follow up. This is in line with previous suggestions that *H pylori* infection would probably play a role in the early events of ACS.^32^ As mentioned, *H pylori* has been shown to cause platelet aggregation.^32^,^47^ During the acute phase of ACS and plaque disruption, this could lead to local inflammation, aggravating platelet aggregation, a crucial event leading to acute myocardial ischaemia. However, these effects would diminish as the plaque becomes stable in the weeks after the ACS. This process may explain why *H pylori* seropositivity was only associated with risk of shortterm outcomes, in spite of varying length of infection among participants.

If a causal relationship between *H pylori* infection and early ACS morbidity and mortality could be established with further prospective studies, this valuable information could be used to improve ACS patients’ survival, hypothetically by treatment of *H pylori* infection in patients presenting with ACS. A previous randomised clinical trial on 325 patients with ACS has shown a significant reduction in incidence of cardiac death or readmission due to ACS as a result of antibiotic treatment for *H pylori* and *Chlamydia pneuminiae*.^18^ However, further prospective studies and randomised trials are needed in order to confirm the role of antibiotic therapy in CAD patients.

As a prospective cohort, this study may suffer from limitations such as unsuspected selection bias or unidentified confounding factors. It has been suggested that *H pylori* seropositivity may reflect the socio-economic status of patients or their early childhood environment,^48^ which may be the actual causes of the observed relationships. However, the present study was carried out on a fairly socially homogenous population, as typical for a relatively small town, and the participants were only recruited from a single hospital, which further identifies them as having a similar socio-economic status. Therefore we suggest it is unlikely that the observed differences were due to socio-economic disparities reflected by *H pylori* seropositivity. Moreover, *H pylori* seropositivity did not show any relationship with other cardiac risk factors among our participants.

## Conclusion

The results of this prospective study provide further evidence that *H pylori* infection could affect the early prognosis in CAD patients. Randomised clinical trials are needed to establish this causal relationship and evaluate the role of antibiotic treatment in these patients.
